# Special Issue “Advanced Signal Processing in Intelligent Systems for Health Monitoring”

**DOI:** 10.3390/s19214727

**Published:** 2019-10-31

**Authors:** Maysam Abbod, Jiann-Shing Shieh

**Affiliations:** 1 Department of Electronic and Computer Engineering, Brunel University London, Uxbridge UB8 3PH, UK; 2 Department of Mechanical engineering, Yuan Ze University, Taoyuan 32003, Taiwan

**Keywords:** health monitoring, artificial intelligence, intelligent system, machine learning, big data, biomedical systems, mechanical systems

## Abstract

Recently, significant developments have been achieved in the field of artificial intelligence, in particular the introduction of deep learning technology that has improved the learning and prediction accuracy to unpresented levels, especially when dealing with big data and high-resolution images. Significant developments have occurred in the area of medical signal processing, measurement techniques, and health monitoring, such as vital biological signs for biomedical systems and noise and vibration of mechanical systems, which are carried out by instruments that generate large data sets. These big data sets, ultimately driven by high population growth, would require Artificial Intelligence techniques to analyse and model. In this Special Issue, papers are presented on the latest signal processing and deep learning techniques used for health monitoring of biomedical and mechanical systems.

## 1. Introduction

Health monitoring is a diverse subject since it covers biological systems as well as physical systems. Biological systems are usually associated with the monitoring of human health, whether it is related to diagnosis of diseases, monitoring of daily activities, or vital signs. On the other hand, physical systems health monitoring is associated with checking physical systems, such as structures and rotary or linear displacement machines. Structural health is related to checking vibration, cracks, or fatigues of materials in buildings, aircrafts, or pipes.

Due to the development of handheld-device technologies, processing power, and sensory accuracy, the penetration into the biomedical field has received intense interest, which has led to the development of new sensors and signal processing algorithms in the field. However, there is still a need to integrate different systems and technologies such that real-time detection and diagnosis can be made available to all people to meet the demand and requisites of the world health monitoring systems [1].

On the other hand, advances in technology, such as high-resolution cameras, optical sensors, drones, and robotics, have evolved the generation of intelligent monitoring systems for physical system health monitoring. Infrastructure health monitoring systems can monitor vibration in buildings, displacement, rotation, stress/strain, cracks/spalling, and defects either directly or remotely. Structural health traditionally is monitored using conventional sensors with data acquisition, transmission, and information processing to assess the structure’s health check. Recently, smart sensors with embedded microprocessors and wireless communication are being used, in addition to fibre optic sensor technology and automated low-cost photogrammetry for flexible structure monitoring [2,3]. Furthermore, non-destructive testing equipment, such as eddy current equipment in pipes and aircraft bodies, have also evolved to become more accurate and reliable due to the developments of intelligent signal processing systems.

On-line monitoring systems have also been used to monitor rotary and displacement machines. The utilisation of vibration-based sensors in addition to image and wireless communication of smart devices, and the use of programmable and web connected applications are the base for the next-generation measurement technology of structural health monitoring. The abundance of handheld smartphones with an easily programmable framework has helped in modifying the relevant software to acquire data using embedded sensors. And in addition, noncontact sensors, such as unmanned aerial vehicles (drones) and mobile sensors, to acquire structural data. The state-of-the-art methods have been presented in a detailed literature review of the recent applications of smartphones, unmanned aerial vehicles (UAVs), cameras, and robotic sensors used for structural condition monitoring and maintenance [4].

The acquired data needs to be filtered, processed, and classified using the latest developments in Artificial Intelligence (AI), such as deep learning and signal processing algorithms like Wavelets transforms (WL) and Empirical Mode Decomposition (EMD). Such algorithms are computationally intensive and require high speed computing. This has caused no major hurdle as the developments and advances in computing has fulfilled this requirement, such as the use of GPUs, computer clusters, edge computing, and cloud applications. Many systems now reside on the cloud, which only requires access via the internet, providing sophisticated algorithm and high-speed processing power.

## 2. Review of the Contributions in This Special Issue

Papers published in this Special Issue can be classified into two groups, human health monitoring (biological systems) and structural health monitoring (physical systems). 

For the field of health monitoring applications to biological systems (human health), this issue included five papers in the field. The use of signal processing and deep learning algorithms is common among the recent applications. A typical example is heart failure detection using R-R interval to monitor the heart rate variability using the long short-term memory deep learning network, which can achieve up to 99% accuracy [5]. This example is extended to utilise wearable sensors that can be used for real-time monitoring of people, athletes, and high-risk patients. This application is of particular interest due to the type of recorded data using sensors on moving parts of the body [6]. Wearable devices, also being used to estimate the blood pressure using a photoplethysmogram (PPG) sensor without the need for cuff and pressure extortion [7]. The advances in deep learning allows extracting features from signals that was not possible using conventional algorithms due to artefacts and noise. This also has been experienced with the use of AI for extracting meaningful signals from electromyography (EMG) measurements [8]. 

The use of deep learning has also improved the accuracy of pneumonia detection and classification using electronic nose. An accuracy of up to 94% can be achieved with the aid of deep learning networks. This application has improved the detection accuracy, in addition to cutting the detection time and avoiding the use of bacterial growth which usually takes days before the treatment can start [9]. 

The second group consists of six papers reporting research work on structural health monitoring, many of which address fault detection on rotational machines. Vibration noisy signals are usually used to diagnose faults on machines; however, due to vibration and electrical noise, the signal recorded from sensory equipment is normally contaminated with noise that is sometimes higher in amplitude to the vibration sensor output. As a result, conventional algorithm classification under experimental conditions may severely degrade the accuracy under noisy environmental conditions, which are ubiquitous in practical industrial applications. A one-dimensional (1-D) denoising convolutional autoencoder (DCAE) [10] and a 1-D convolutional neural network (CNN) proved to be excellent solutions to address this problem [11], whereby the former is used for noise reduction of raw vibration signals and the latter for fault diagnosis using the de-noised signals. The DCAE model can be trained using noisy data for learning. Such configuration is used for fault diagnosis of ball-bearing failure in rotational machines [12], railways, and diesel engine operations [13].

In addition to the use of deep learning algorithms, other feature extraction algorithms can be used for pre-processing the signals before classification, such as EMD and WL [14]. This is in addition to other different techniques, such as the use of video images in analysing the movements of large structures [15].

The last paper is a mini review that addresses autonomous health monitoring systems. Autonomous systems are defined within the context of intelligent systems that can learn, adapt, and have a certain degree of self-control when integrated with advanced resources, such as smart sensors and internet access, and can handle big data using AI and deep learning. Such systems should be able to deal with dynamic environments where changes to internal parameters, and disturbances, can be accommodated during operation by self-reconfiguration to adapt to the new environment. A classic example of such system is adaptive control where the control law is adapted according to changes in the environment. However, for autonomous systems, it should be able to reconfigure itself when sensors or actuators fail or become out of their operational range [16].
